# Mapping and understanding the decision-making process for providing nutrition and hydration to people living with dementia: a systematic review

**DOI:** 10.1186/s12877-020-01931-y

**Published:** 2020-12-02

**Authors:** Kanthee Anantapong, Nathan Davies, Justin Chan, Daisy McInnerney, Elizabeth L. Sampson

**Affiliations:** 1 Marie Curie Palliative Care Research Department, Division of Psychiatry, University College London, London, UK; 2 Department of Psychiatry, Faculty of Medicine, Prince of Songkla University, Hat Yai, Thailand; 3 Centre for Ageing Population Studies, Research Department of Primary Care and Population Health, University College London, London, UK; 4 Barnet Enfield and Haringey Mental Health Trust Liaison Team, North Middlesex University Hospital, Sterling Way, London, UK

**Keywords:** Aging, Alzheimer’s disease, Dementia, Decision making, Dehydration, Feeding methods, Nutrition, Systematic review

## Abstract

**Background:**

This systematic review aimed to explore the process of decision-making for nutrition and hydration for people living with dementia from the perspectives and experiences of all involved.

**Methods:**

We searched CINAHL, the Cochrane Library, EMBASE, MEDLINE and PsycINFO databases. Search terms were related to dementia, decision-making, nutrition and hydration. Qualitative, quantitative and case studies that focused on decision-making about nutrition and hydration for people living with dementia were included. The CASP and Murad tools were used to appraise the quality of included studies. Data extraction was guided by the Interprofessional Shared Decision Making (IP-SDM) model. We conducted a narrative synthesis using thematic analysis. PROSPERO registration number CRD42019131497.

**Results:**

Forty-five studies were included (20 qualitative, 15 quantitative and 10 case studies), comprising data from 17 countries and 6020 patients, family caregivers and practitioners. The studies covered a range of decisions from managing oral feeding to the use of tube feeding. We found that decisions about nutrition and hydration for people living with dementia were generally too complex to be mapped onto the precise linear steps of the existing decision-making model. Decision-making processes around feeding for people living with dementia were largely influenced by medical evidence, personal values, cultures and organizational routine. Although the process involved multiple people, family caregivers and non-physician practitioners were often excluded in making a final decision. Upon disagreement, nutrition interventions were sometimes delivered with conflicting feelings concealed by family caregivers or practitioners. Most conflicts and negative feelings were resolved by good relationship, honest communication, multidisciplinary team meetings and renegotiation.

**Conclusions:**

The decision-making process regarding nutrition and hydration for people living with dementia does not follow a linear process. It needs an informed, value-sensitive, and collaborative process. However, it often characterized by unclear procedures and with a lack of support. Decisional support is needed and should be approached in a shared and stepwise manner.

## Background

In 2016 around 44 million people worldwide had dementia [1]. This figure will reach 76 million in 2030 and 135 million in 2050, mainly due to population ageing [2]. As dementia progresses, people experience cognitive, psychological, behavioral, sleep and physical problems. The progression of dementia may cause swallowing difficulties, loss of appetite, inability to recognize food and utensils, difficulties in attention and problems with maintaining a normal eating routine [3]. These can lead to aspiration pneumonia, malnutrition, weight loss, skin breakdown, poor wound healing, and increased confusion [4]. A range of strategies can be used to support eating and drinking difficulties including food modification, dining environment modification, social support for eating and drinking, and behavioral interventions [5].

Artificial nutrition and hydration (ANH) is sometimes offered via invasive procedures such as a nasogastric tube, percutaneous endoscopic gastrostomy (PEG), intravenous hydration and hypodermoclysis (subcutaneous fluid infusion). The evidence of the effectiveness of these interventions is limited, and they can have a negative effect on the wellbeing of the person with dementia [6, 7]. Alternatively, for people with severe dementia, comfort feeding only (CFO) can be offered to provide food and fluids orally to people living with dementia (PLWD) with the goal of comfort and pleasure [8].

Decisions regarding nutrition and hydration for PLWD are often left to family caregivers and practitioners. They may not know the preferences of the person with dementia [9], which cannot be inferred from their preferences regarding other decisions, such as Do-Not-Resuscitate [10]. These decisions are complex and emotive [11]. Challenges with decision-making processes regarding nutrition and hydration may detract from person-centred care, and cause distress to PLWD, family caregivers and practitioners.

Ideally, decisions should be made adopting a shared decision-making approach in which PLWD and family caregivers collaborate with healthcare practitioners [12]; however, concepts of shared decision-making are still inconsistently applied in dementia care [13]. Currently, there are no studies that have investigated the stepwise process of making decisions about nutrition and hydration for PLWD. Existing work focuses only on specific steps, rather than understanding the process as a whole to identify key areas where support is needed and for whom [14].

This systematic review aims to understand the steps in the decision-making process regarding nutrition and hydration for PLWD from the perspectives and experiences of all involved. The specific review questions are:

1. What are the key decisions that need to be made about nutrition and hydration for PLWD?
2. What are the steps required for making decisions made by PLWD, caregivers and practitioners and how do these steps map onto an existing decision-making model?
3. What are the facilitators and barriers to making decisions?

## Methods

### Design

A systematic review of quantitative studies, qualitative studies and case studies was conducted using a narrative synthesis [15]. We followed the Preferred Reporting Items for Systematic Reviews and Meta-Analysis (PRISMA) Statement in reporting the review [16] (see Additional file 1). The review protocol is registered on Prospero (CRD42019131497).

### Criteria for inclusion

We included peer-reviewed original research articles reporting the decision-making process regarding nutrition and hydration for PLWD fulfilling the following criteria in Table 1:

**Table 1:** Inclusion criteria for eligible studies

Inclusion criteria	
- **Population:** At least 80% of the study participants were PLWD, informal and formal caregivers of PLWD, and/or practitioners caring for someone with dementia. This cut-off was based on proportion of PLWD among nursing home residents [17] and used by other published studies in Cochrane [18]. - **Data:** Decision-making process regarding nutrition and hydration interventions for PLWD; determinants, facilitators and barriers of the decisions - **Intervention:** Any nutrition and hydration interventions, for example, oral- or hand-feeding, and enteral or parenteral ANH - **Setting:** All settings in which decisions regarding nutrition and hydration for PLWD were made. - **Study design:** Quantitative study, qualitative study, case study (case studies were included, because we expected that we could gain more insights into the whole decision-making experiences at an individual case.)	

We excluded reviews, letters, opinion pieces, conference abstracts, and theses. Due to resource limitations, studies not published in English were excluded. There was no restriction on publication year. During the pilot screening and through team discussions, we developed a detailed guide for study selection (see Additional file 2).

### Search strategy

We searched the following electronic databases of peer-reviewed articles: CINAHL, the Cochrane Library, EMBASE, MEDLINE and PsycINFO. We tailored search strategies for each database, using a combination of Medical Subject Heading (MeSH) and keywords. The search was initially guided by existing Cochrane systematic reviews on enteral feeding for PLWD [7] and shared decision-making in other diseases [19]. Initial scoping of the literature helped to refine the search terms, identify synonyms and abbreviations (see Additional file 3). We tracked citations, searched reference lists, conducted additional hand searches and consulted experts in the field. The search was carried out from inception of each database until 29 January 2020.

### Selection procedure

**Fig. 1:**
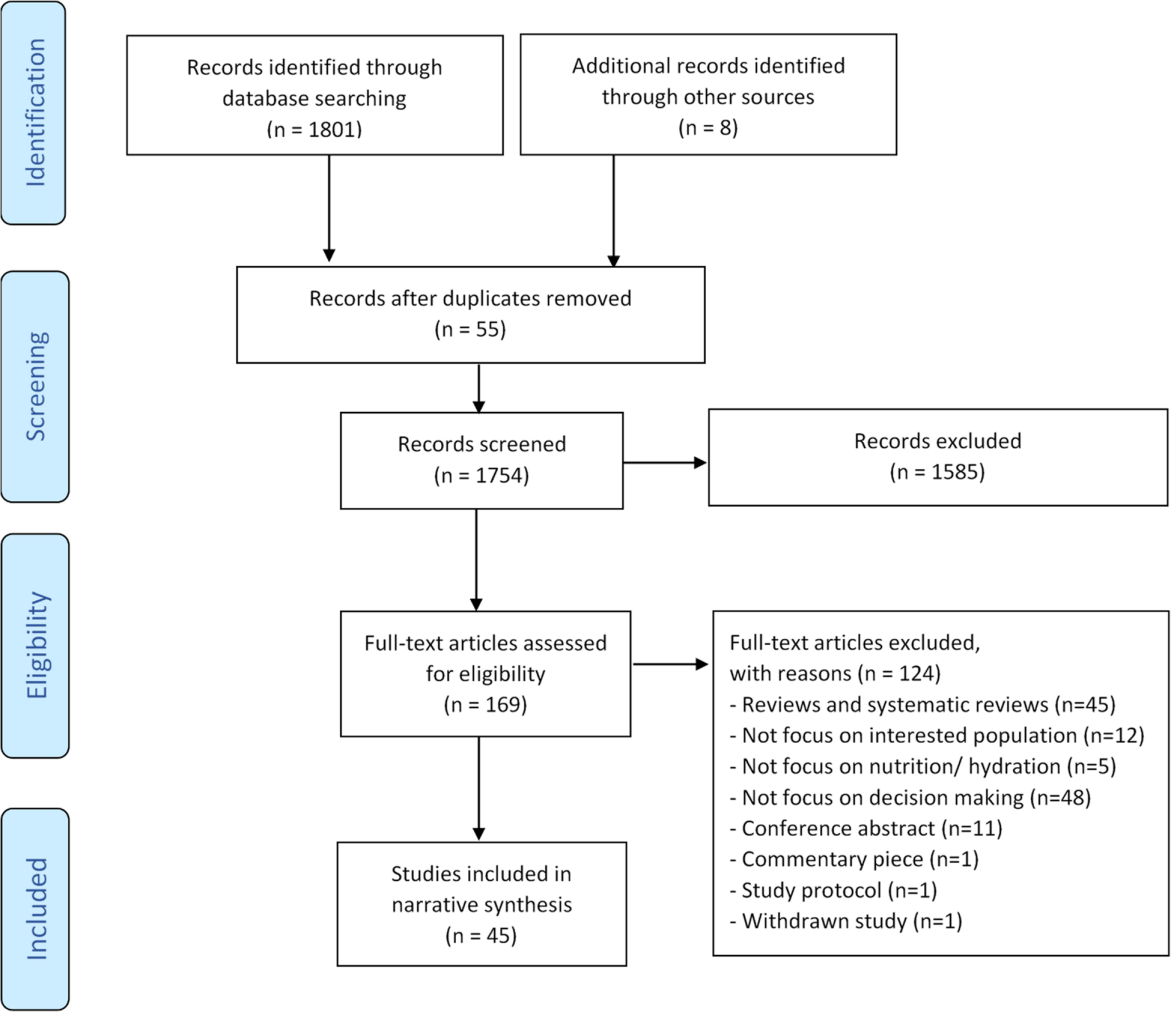
PRISMA flow chart of study selection

All article titles and abstracts were screened against the inclusion/exclusion criteria by at least two reviewers (KA, JC, DM). Articles considered potentially relevant were read in full by one author (KA). A random sample (35%) of full texts was checked by a second reviewer (JC or DM). Disagreements were discussed with a third reviewer (ND or ELS) to reach consensus. Figure 1 shows the PRISMA flowchart.

### Quality appraisal

We used the Critical Appraisal Skills Programme (CASP) toolkit of quality appraisal tools to appraise the quality of studies [20]. We used a tool proposed by Murad and colleagues for evaluating the methodological quality of case studies [21]. We appraised the methodological quality of the included studies against the criteria in the selected tools and rated each study as good, moderate, or poor. Studies judged to be of higher quality were prioritized in the results and discussion, but no studies were excluded based on quality. Quality assessment was led by one author (KA), and a random sample (10%) of included studies was checked by a second reviewer (ND or ELS).

### Data extraction

We used the eight key steps of the Inter-Professional Shared Decision Making (IP-SDM) model [22] to guide data extraction of key components of the decision-making process. We chose this model because it focuses on both a stepwise framework and shared approach of decision-making process for healthcare issues. It moves beyond a single patient-doctor dyad to explain an interprofessional approach and family involvement. The model describes decision-making factors at three different system levels: micro (individuals including PLWD, family caregivers, individual practitioners), meso (healthcare teams within organizations) and macro (broader policies and social context). It consists of eight steps: identification of the decision to be made, information exchange between individuals making the decision, exploration of values and preferences of those involved, feasibility of options, consideration of preferred choices, deliberation of an actual decision, implementation of the actual decision and outcome evaluation. We also used the Ottawa Decision Support Framework (ODSF) model to guide and extract data regarding decision factors, facilitators and barriers regarding nutrition and hydration [23].

Data from included studies were extracted and recorded in a standardized data extraction table including study characteristics, participant characteristics, common nutrition and hydration decisions, decision-making process (aligned to IP-SDM), decision factors (aligned to ODSF), and sources of funding of the included studies (see Additional file 4). This was piloted on three papers by three reviewers (KA, JC, DM). Any discrepancies were discussed with a third reviewer (ND or ELS) to reach consensus. Data extraction of the remaining papers was completed by one author (KA).

### Synthesis

We conducted a narrative synthesis using thematic analysis and tabulation following guidance from Popay and colleagues [15], which was led by KA. This thematic analysis had three stages: 1) coding the extracted data within the data extraction table of the eight steps of IP-SDM model; 2) generating themes/subthemes from the coded data within each step; and 3) mapping and revising the IP-SDM model based on the generated themes/subthemes. We also extracted and noted alternative perspectives within the data and included them in the synthesis. Three reviewers (KA, JC, DM) independently coded two papers, followed by a meeting to discuss the coding and devise an agreed coding frame. One author (KA) then coded the remaining papers using the agreed coding frame. Themes, data synthesis and revisions to the model were discussed iteratively among reviewers (KA, ND, ELS).

## Results

### Description of included studies

Forty-five articles met the eligibility criteria. Twenty qualitative, 15 quantitative and 10 case studies were included. Included papers were published between 1987 and 2019 and comprised data from 17 countries. Over 40% (19/45) of the included studies were conducted in the USA and more than half (23/45) were conducted in Europe. The most frequent settings were nursing homes (25/45), and acute hospitals (22/45).

Studies involved at least 6020 patients, caregivers and practitioners: 272 PLWD; 3051 physicians; 1929 nurses and allied healthcare professionals; 740 family caregivers; 28 professional surrogates. However, due to some studies using direct observational methods, the precise number and type of participants could not be determined. A smaller number of PLWD was involved in some included studies using direct observational methods; their perspectives were not always represented in the included studies (Table 2).

**Table 2:** Characteristics of included studies

First author (year)	General study aim	Study design/ methods	Setting (Country)	Main feeding methods being studied	Population of the study	Number of participants (staff:surrogate)	Quality rating
**Quantitative studies**							
Hodges MO (1994) [24]	Examine internists’ attitudes and decision-making regarding tube feeding for older patients including people living with dementia (PLWD) with unknown patient and family’s preferences	Self-administered, structured questionnaire with case scenarios	Nursing home (USA)	Tube feeding in general	Physician: Board-certified Internal Medicine (99%), subspecialty (55%) (no raw number of the subgroups)	326 (326:0)	Good
Kuehlmeyer K (2015) [25]	Explore how nursing staff evaluate the nonverbal feeding related behaviors of PLWD	Self-administered, structured questionnaire	Nursing home (Germany)	Unspecified: nonverbal feeding-related behaviors towards tube feeding and hand feeding	Mixed: certificated nurse (65%), nursing assistant (23%) (no raw number of the subgroups)	131 (131:0)	Good
Teno JM (2011) [26]	Examine tube feeding decision-making based on interviews with bereaved family carers	Telephone interviews with structured questionnaire	Mixed- PLWD died in nursing home (76.4%), hospital (15.6%) (USA)	Tube feeding in general	Family member: child of the decedent = 66.6%, spouse = 8.4%, sibling = 3.5%, other = 21.5%	486 (0:486)	Good
Chen PR (2019) [27]	Explore perceptions of hospital staff regarding reducing tube feeding use of patients with advanced dementia	Self-administered, structured questionnaire	Acute Hospital (Taiwan)	Tube feeding in general	Mixed: Physician (101), Nurse (278), dietician (42), paramedial staff (pharmacists, speech therapists, occupational therapists, physical therapists, psychologists, and respiratory therapists) (103), administrative staff/ social worker (44), attendant/ volunteer (56)	624 (624:0)	Moderate
Gieniusz M (2018) [28]	Evaluate physician knowledge and perceptions regarding the use of percutaneous endoscopic gastrostomy (PEG) tubes in PLWD	Self-administered, structured questionnaire	Acute hospital, outpatient (USA)	PEG	Physician: attending physician (82), resident physician (50), fellow (11), others (3); no information of the rest	168 (168:0)	Moderate
Kwok T (2007) [29]	Examine attitudes of family carers of PLWD regarding life sustaining treatment including tube feeding	Interview with structured questionnaire	Mixed- nursing home (84% of PLWD under their care), psychogeriatric ward, long-term care ward (Hong Kong)	Nasogastric (NG) intubation, PEG	Family member: spouse (9), offspring (32), other (10)	51 (0:51)	Moderate
Modi SC (2007) [30]	Examine the relationship between race of patient/ physician and recommendation for PEG placement	Self-administered, structured questionnaire with case scenarios	Acute hospital, follow-up clinic in hospital (USA)	PEG	Physician: Family Medicine (457), Internal Medicine (479), Geriatrics (44), unknown (103)	1083 (1083:0)	Moderate
Norberg A (1994) [31]	Compare nurses’ reasons to feed or not to feed PLWD within six countries	Interview with structured questionnaire and case scenarios	Mixed- institutions considered providing high quality care (Australia, Canada, China, Finland, Israel, Sweden, USA)	Unspecified: hand feeding, forced feeding, tube feeding	Nurse: ward sister (67), staff nurse (82); participants from USA (39), Australia (20), Canada (20), China (8), Finland (20), Israel (20), Sweden (20)	149 (149:0)	Moderate
Pasman HRW (2004) [32]	Examine characteristics of PLWD for whom it is decided to forgo artificial nutrition and hydration (ANH) and characteristics of decision-making process	Self-administered, structured questionnaire	Nursing home (Netherlands)	ANH in general: PEG, NG, intravenous (IV) infusion, subcutaneous hydration (hypodermoclysis)	Patient: 178 cases (PLWD) in whom ANH was forgone; questionnaire about the cases filled by nursing home physician (178 cases), nurse (128), family member (128) - filled by all (116 cases)	178 (0:0) (178 PLWD) *unit of analysis is PLWD	Moderate
Pengo V (2017) [33]	Examine physicians and nurses’ opinions regarding antibiotics, artificial nutrition and hydration for PLWD with different life expectancies	Self-administered, structured questionnaire	Mixed- hospital, geriatric clinic, residential and semi- residential facilities (Italy)	ANH in general	Mixed: physician (288), nurse (763)	1051 (1051:0)	Moderate
Shega JW (2003) [34]	Examine factors that affect physician recommendations of PEG for PLWD	Self-administered, structured questionnaire with case scenarios	Acute hospital: case scenarios of PLWD admitted to an acute hospital (USA)	PEG	Physician: General Internal Medicine = 50.8%, Family practice = 49.2%	195 (195:0)	Moderate
Valentini E (2014) [35]	Examine physicians and nurses’ opinions regarding ANH for terminally ill PLWD	Self-administered, structured questionnaire	Mixed- hospital, Geriatric clinic, residential and semi- residential facilities (Italy)	ANH in general	Mixed: physician (288), nurse (763)	1051 (1051:0)	Moderate
van Wigcheren PT (2007) [36]	Examine incidence of ANH in PLWD and characteristics of ANH decision-making process for PLWD	Self-administered, structured questionnaire	Nursing home (Netherlands)	ANH in general: food and fluids (NG, PEG) and fluids only or hydration (IV infusion, hypodermoclysis)	Physician: nursing home physician	704 (704:0)	Moderate
Babiarczyk B (2014) [37]	Explore attitudes and experiences of caring staff about feeing problems	Self -administered, structured questionnaire	Nursing Home (Norway, Poland)	Unspecified: feeding difficulties, forced feeding	Mixed: professional staff (nurses (19), certificated nurse assistant (10), healthcare assistant (8) nursing student (2), Dietician (1), physiotherapist (1)), Unprofessional (student (3), assistant (8)); participants from Norway (28), Poland (24)	52 (52:0)	Poor
Golan I (2007) [38]	Evaluate decision-making process of family members and physicians regarding PEG insertion for PLWD referred for PEG	Interview with structured questionnaire	Acute hospital (Israel)	PEG	Mixed: physician referring for PEG (72), family member or guardian (126), gastroenterologist (34)	232 (106:126)	Poor
**Qualitative studies**							
Aita K (2007) [39]	Explore why Japanese physicians feel bound to provide ANH, particularly PEG, to PLWD	Semi-structured interviews	Mixed- acute hospital, long-term care hospital (Japan)	ANH in general: particularly to PEG	Physician: Internal Medicine (11), Surgery (2), Neurology (4), Neurosurgery (3), Palliative care (1), Psychiatry (3), Geriatrics (1), GI surgery (1), GI Internal Medicine (1), Family physician (2), General Medicine (1)	30 (30:0)	Good
Bryon E (2010) [40]	Explore nurses’ involvement in ANH decision-making for hospitalized PLWD	Semi-structured interviews	Acute hospital ward- Geriatrics, Geriatric Psychiatry, Palliative support team, Internal Medicine (Belgium)	Tube feeding, gastrostomy	Nurse: registered nurse (17) Master’s in Nursing Science (2), undergraduate nurse (2)	21 (21:0)	Good
Bryon E (2012) [41]	Explore nurses’ experiences in ANH decision-making for hospitalized PLWD	Semi-structured interviews	Acute hospital ward- Geriatrics, Geriatric Psychiatry, Palliative support team, Internal Medicine (Belgium)	Tube feeding, gastrostomy	Nurse: registered nurse (17) Master’s in Nursing Science (2), undergraduate nurse (2)	21 (21:0)	Good
Bryon E (2012) [42]	Explore nurses’ experiences with nurse-physician communication during ANH decision-making for hospitalized PLWD	Semi-structured interviews	Acute hospital ward- Geriatrics, Geriatric Psychiatry, Palliative support team, Internal Medicine (Belgium)	Tube feeding, gastrostomy	Nurse: registered nurse (17) Master’s in Nursing Science (2), undergraduate nurse (2)	21 (21:0)	Good
Jox RJ (2012) [43]	Explore experiences of family and professional surrogates regarding medical decisions including tube feeding for PLWD	Think aloud with case scenarios	Unspecified (Germany)	PEG	Mixed: family surrogate (16), professional surrogate (16)	32 (0:32)	Good
Lopez RP (2010) [44]	Explore organizational influence on practice of tube feeding for PLWD in nursing homes	Focused ethnographic; observations, semi-structured interviews, abstraction of publicly available material	Nursing home- two nursing homes with high and low use rate of tube feeding (USA)	Unspecified: feeding practice regarding both tube feeding and hand feeding	Mixed: observations of all nursing home staff and residents (no number); semi-structured interviews of staff including director of nursing (2), senior administrator (2), speech and language pathologist (2), licensed nurse (11), certified nurse assistant (6), social worker (2), diet technician (2), recreational therapist (2)	At least 29 (29:0)	Good
Luhnen J (2017) [45]	Explore values and experiences of legal representatives of PLWD regarding healthcare decisions including PEG	Semi-structured interviews	Mixed- associations related to legal representatives and nursing homes (Germany)	PEG	Mixed: family surrogate (12), professional surrogate (12)	24 (0:24)	Good
Pasman HRW (2003) [46]	Explore nurses’ experiences and responses to feeding problems of PLWD in daily practice	Observations, semi-structured interviews	Nursing home (Netherlands)	Unspecified: hand feeding, forced feeding, ANH	Mixed: observations of 94 PLWD needed help with meals; more depth for those 60 PLWD having feeding problems and 15 having aversive behavior; including their family and 46 nurses helping them	At least 140 (46:0) (94 PLWD)	Good
Pasman HRW (2004) [47]	Explore role and influence of participants (family and professionals) in the decision making to start or forgo ANH for PLWD	Observations, semi-structured interviews	Nursing home (Netherlands)	ANH in general: to start or forgo ANH	Mixed: observations of decision-making process for 35 PLWD; involving nursing home physician (8), family members (32), nurses (43)	83 (51:32)	Good
The AM (2002) [48]	Explore decision-making process behind withholding ANH from PLWD in nursing home	Observations, semi-structured interviews	Nursing home (Netherlands)	ANH in general: withholding ANH	Mixed: observations of 35 candidates (PLWD) for the withholding of ANH; involving Nursing home physician (8), family members (32), nurses (43)	83 (51:32)	Good
Buiting HM (2011) [49]	Explore Dutch and Australian doctors’ experiences of decision-making of ANH for PLWD	Semi-structured interviews	Mixed- nursing home, hospital (Australia, Netherlands)	ANH defined by the participants themselves	Physician: nursing home physician (14), geriatrician (6) GP (9) palliative care specialists (1); participants from Netherlands (15), Australia (15)	30 (30:0)	Moderate
Gil E (2018) [50]	Explore family guardians’ attitudes and cultural considerations of decision-making of tube feedings for PLWD	Observations, follow-up semi-structured interviews	Acute hospital- Gastroenterology outpatient unit (Israel)	PEG	Family member: descent (15), sibling (2)	17 (0:17)	Moderate
Lopez RP (2010) [51]	Explore nurses’ beliefs, knowledge, and roles in feeding decisions for PLWD	Semi-structured interviews	Nursing home (USA)	Unspecified: feeding decisions towards both tube feeding and hand feeding	Nurse: licensed practical nurse (6) Registered nurse (5)	11 (11:0)	Moderate
Jansson L (1992) [52]	Elucidate nurses’ ethical reasoning and decision-making of forced feeding for PLWD with refusing-like behaviors	Semi-structured interviews with case scenarios	Mixed- nursing home, psychogeriatric clinics, somatic long-term clinic (Sweden)	Forced feeding for PLWD with refusal-like behaviors	Nurse: all registered nurses	20 (20:0)	Poor
Nagao N (2008) [53]	Explore American and Japanese experts’ ethics consultation focusing nutritional management for PLWD	Semi-structured interviews with case scenarios	Acute hospital (Japan, USA)	ANH in general: NG, PEG, IV	Mixed: US psychiatrist (1), Japanese Internal Medicine (1), US and Japanese ethicist (2); participants from USA (2), Japan (2)	4 (4:0)	Poor
Norberg A (1987) [54]	Explore nurses’ experiences of withdrawing and withholding nutrients and fluids from PLWD and interpret their reasons regarding ethical principles	Semi-structured interviews with case scenarios	Nursing home (Sweden)	Unspecified: forced feeding, tube feeding, infusion, active euthanasia	Nurse: registered nurse (14), practical nurse (17), mental nurse (10), nurses’ aid (19)	60 (60:0)	Poor
Norberg A (1987) [55]	Explore healthcare professionals’ attitudes towards feeding of PLWD	Semi-structured interviews, focused group	Mixed- long-term care institutional services, nursing home, psychogeriatric hospital (Israel)	Unspecified: forced feeding, tube feeding, infusion, active euthanasia	Mixed: individual interviews of physician (10), social worker (1), nurse (16), nurses’ aid (3); Group interviews of 4–15 people each group (no exact number) including psychologist; at least 60 participants	60 (60:0)	Poor
Pang MCS (2007) [56]	Explore cultural influence on tube feeding decisions for PLWD in USA and Hong Kong	Observations	Specialized long-term care unit in hospital (Hong Kong, USA)	Tube feeding in general: to or not to forgoing tube feeding	Mixed: observations of PLWD, family member, healthcare professional	No information about number of participants	Poor
Smith L (2016) [57]	Explore nurses’ perceptions and beliefs about suffering regarding ANH for PLWD	Focused group with case scenarios	Home care for people with late stage dementia (USA)	ANH in general: suffering from ANH	Nurse: home healthcare nurse	17 (17:0)	Poor
Wilmot S (2002) [58]	Explore how nursing staff apply ethical principles in feeding problems of PLWD	Focused group with case scenarios	Acute hospital: wards in a psychiatric hospital (UK)	Unspecified: feeding problems with a spectrum of methods (ANH and hand feeding)	Mixed: nurse, health care assistant staff	12 (12:0)	Poor
**Case studies**							
Berger JT (1996) [59]	Describe conflict between staff and family over differing assessments of resident’s quality of life and the cultural context of illness	Case study	Nursing home (USA)	ANH in general: NG, permanent gastrostomy	Case study involved daughter, physician, nursing staff	NA	Good
Christenson J (2019) [60]	Describe ethical dilemma concerning stop hand feeding in people with advanced dementia	Case study	Hospice (USA)	Hand feeding in general; assisted hand feeding, presumed wishes of ‘voluntary stopping eating and drinking’ (VSED), consider comfort feeding only (CFO)	Case study involved wife, physician, nurse, unlicensed assistive personnel, hospice’s administration, partner organization of the hospice	NA	Good
Meier CA (2015) [61]	Describe ethical dilemma of withholding food and drink in a patient with advanced dementia.	Case study	Hospice, nursing home (USA)	Hand feeding in general: fully assisted hand-feeding, verbally expressed of VSED, CFO	Case study involved daughter, nurse, social worker, chaplain, hospice medical director, nursing home director	NA	Good
Orr RD (1991) [62]	Describe clinical and ethical analysis of decision-making regarding tube feeding for PLWD	Case study	Acute hospital, nursing home (USA)	Surgical placement of gastrostomy	Case study involved daughter, nursing home staff, attending physician, director and administrator of nursing home, ethics consultant	NA	Good
Orr RD (2002) [63]	Describe the ethics consultation and decision-making process regarding tube feeding for PLWD	Case study	Acute hospital, nursing home (USA)	Unspecified: tube feeding, gastrostomy, IV, total parenteral nutrition (TPN), time-trial, CFO	Case study involved daughter, physician, bedside nurse, ethics consultant	NA	Good
Tapley M (2014) [64]	Describe decision-making process regarding tube feeding for PLWD in the best interests meeting	Case study	Nursing home (UK)	Tube feeding: radiologically inserted gastrostomy (RIG) tube	Case study involved husband, two daughters with conflicting opinions, specialist dementia nurse, GP, nursing home manager, staff nurse, dietician	NA	Good
Back AL (2005) [65]	Describe conflicts between physicians and family and a step-wise approach to deal with the conflicts	Case study	Nursing home (USA)	ANH in general: NG, IV, considering PEG	Case study involved husband, medical director of nursing home (physician)	NA	Moderate
Clibbens R (1996) [66]	Describe a situation where the patient’s difficulties in swallowing became an ethical dilemma for family and the author	Case study	Acute hospital, nursing home (UK)	NG	Case study involved daughter, nurse, hospital team	NA	Poor
Hodges MO (1994) [67]	Describe and discuss ethical issues in tube feeding decisions for older people including the case of PLWD	Case study	Nursing home (USA)	NG with considering soft patient restraints	Case study involved sorority friend, nursing home physician, nurse, dietician	NA	Poor
Scarpinato N (2000) [68]	Describe the author’s decision making and uncertainty	Case study	Acute hospital, nursing home (USA)	PEG	Case study involved attending physician, nurse, niece (never contact before)	NA	Poor

### Quality appraisal

Most included studies had poor (*n* = 12) to moderate (*n* = 14) quality (see Additional file 5). Articles rated as poor and moderate-quality showed selection bias, non-respondent bias, misclassification bias and potential confounding effects. Most studies used self-administered questionnaires and face-to-face interviews and were subject to recall bias and response bias. Ten studies used hypothetical scenarios to initiate and stimulate discussion. Six studies used direct observations of the actual decision-making process; however, they were still subject to attention bias and subjective interpretation by observers.

### Narrative synthesis: decision-making process

**Fig. 2:**
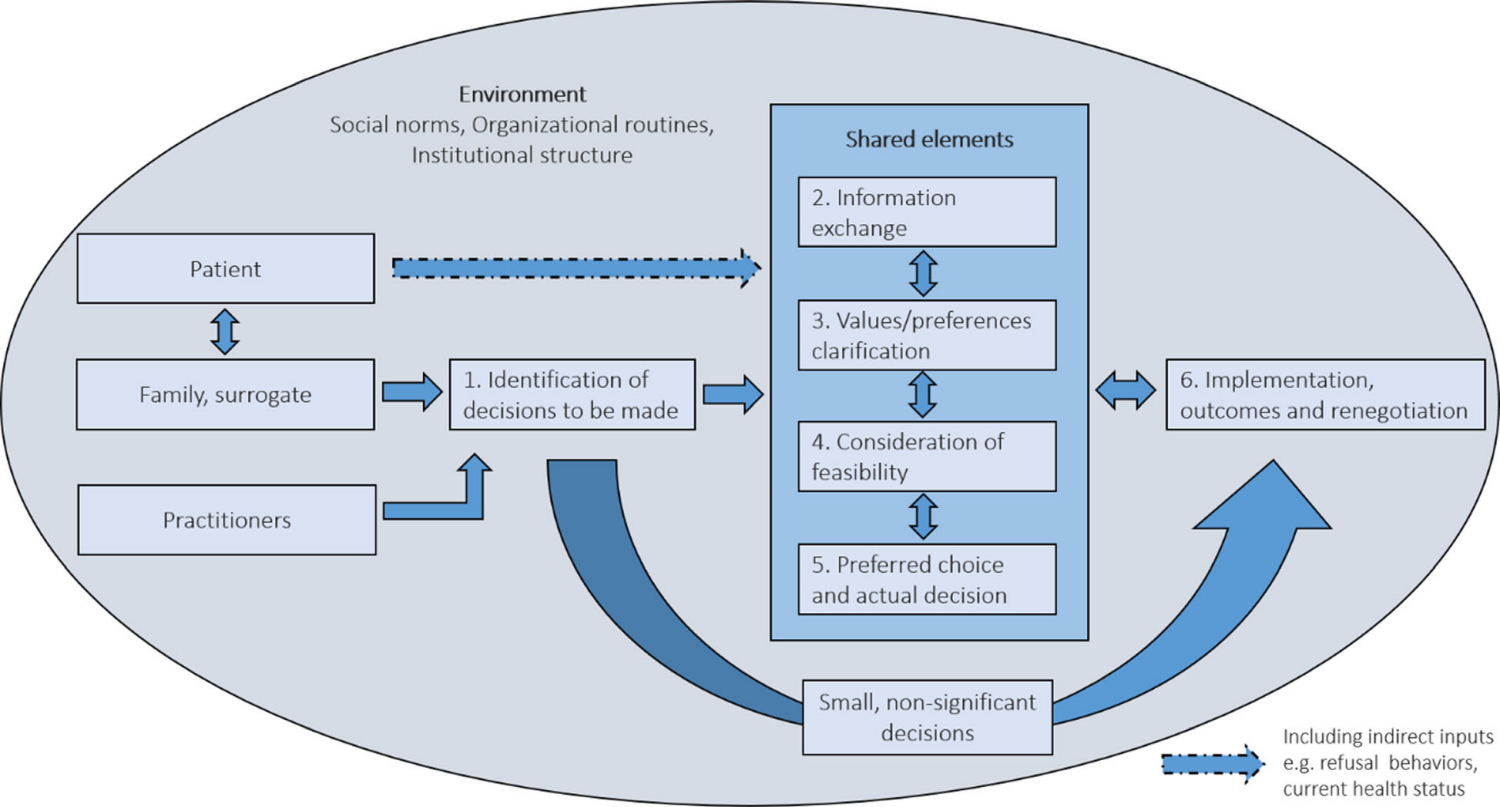
Diagram shows the decision-making process of nutrition and hydration for people living with dementia

We ordered the themes according to the IP-SDM model [22]. During analysis, we recognized that two categories within this model appeared to be closely related. We therefore chose to revise the model, reducing it from eight to six steps by combining a) the consideration of preferred choices with deliberation of an actual decision and b) the implementation of the decision with outcome evaluation (see Fig. 2). Emerging themes are presented following each of the six steps of the model.

#### Identification of decision to be made

Across the studies, key decisions about nutrition and hydration that needed to be made for PLWD concerned various eating and drinking problems, for example, the inability to recognize food, food refusal behaviors, chewing and swallowing difficulties, recurrent chocking and aspirations, changes in alertness, and significant weight loss. These occurred either during acute illness or because of the progression of dementia [48]. The decisions to be made were focused on whether and how to start or forgo ANH, to continue or stop hand-feeding, and to consider CFO.

- Decision not using shared approach

Nurses and family caregivers often encountered day-to-day decisions of eating problems in PLWD showing progressive decline. They had to make the decisions alone or sometimes with colleagues present at the time. These day-to-day situations frequently required an immediate decision to be made such as whether to continue encouraging the person to eat if they refused at each meal [46, 58]. Thus, formal discussion was often not possible, or appropriate.

- Decision using shared approach

In the context of acute illness, family caregivers and practitioners were likely to have discussions and consider using ANH, especially in acute infections [26, 32, 36], as they considered dehydration in acute illness as treatable. However, they felt more reluctant to start ANH if the overall health of the patient was poor or if they were considered to be in the end-stages of dementia [48]. Family caregivers may request practitioners to stop hand-feeding a person with dementia who had previously expressed views that they did not want food or fluids when they became “terminally ill”. The decision to stop hand-feeding was challenging if the person no longer had decisional capacity, but still accepted food or fluids [60, 61].

Across all decisions, assessments of mental capacity and swallowing were important. This included considering other causes such as depression [45, 48, 68] and clarification of the goals of care for the patients, which usually aimed to promote comfort [27, 44, 48, 49, 67] .

- Initiator of the shared decision-making process

The person initiating the decision process was likely to be a person who spent most time with the person with dementia and noticed the changes, usually nurses [40, 41, 44, 47] and sometimes family caregivers [61]. Nurses raised the problem to a physician or nursing home director during a team meeting, sometimes by informal conversations [47, 51] and together with family caregivers [47]. However, nurses might end their involvement after the notification, as they perceived that their role was to convey the message and guide communication [40].

#### Information exchange

During the decision-making process, information about the person’s conditions and feeding interventions was shared among PLWD, caregivers and practitioners. This process was biased by their understanding and emotions towards the situation and interventions.

- Understanding of disease and interventions

We identified diverse interventions in this review, spanning from regular oral feeding to the use of tube feeding, which may lead to reduced pleasure in eating and social contact [58]. The level of knowledge, preferences and attitudes towards these interventions varied among all involved. Several studies found that family caregivers and practitioners had poor understanding and unrealistic expectations of tube feeding for PLWD [27–29, 34, 37, 38, 51, 56]. Around 60% of physicians also underestimated the 30-day mortality rate found for PLWD following PEG insertion [28, 34]. Physicians reported that understanding poor outcomes and irreversibility in advanced dementia were the most important factors influencing provision of ANH [24, 32]. If patients were not considered imminently dying, practitioners and family members might consider ANH [33, 48, 62, 63].

- Explaining the disease and intervention

Insufficient information regarding feeding interventions was provided for family caregivers [26, 38]. Practitioners tended to give biased information in favor of tube feeding, if they feared aspiration from hand-feeding or that the persons were suffering from inadequate nutrition and hydration [27, 44, 47]. However, in many other cases the family was emotionally unable to assess the information and needed more time to accept the current situation, especially the approaching death of the person with dementia [47, 48]. Sixty-three percent of physicians reported that family requested PEG for the person even when the physician explained they would not recommend it [28, 50]. In many cases, practitioners felt the need to guide family caregivers to understand the seriousness of patients’ condition, discouraging them from using tube feeding by explaining their unrealistic expectations of the benefits [40].

Information about clinical outcomes of the feeding interventions alone did not have a significant influence on confidence or comfort in making decisions [29]. Therefore, information to be considered in this context included information about the person’s life history, age, experiences with other family members, and the wishes and opinions of the person with dementia, family caregivers and practitioners [39, 40, 43, 45, 52]. The person’s current wellbeing also influenced decisions among practitioners, for example, physicians were less likely to oppose the initiation of tube feeding, if the patients generally looked happy and were not restrained [24, 34, 43, 67].

- Recognizing the emotions of all involved

Uncertainty around disease progression, lack of knowledge and confusing roles regarding the decision-making process created feelings of guilt, exclusion and frustration, and sometimes led to conflicts among family caregivers, healthcare practitioners and nursing home staff [41, 51, 54, 56, 59, 65, 66]. Regular discussions, open team meeting and family meetings to build trust and share key information facilitated the process [42, 45, 46, 58, 59, 65]. However, it was sometimes necessary to consult an ethics committee or specialist to resolve conflicts [62, 64].

#### Values and preferences clarification

Depending on their role, decision-makers had varying perceptions of the necessities of feeding interventions, and approached the decisions in different ways. Personal preferences and social values played an important role in the differences.

- Value and preferences regarding feeding interventions

ANH was generally viewed as a medical intervention that could unnecessarily prolong the person’s life [24, 32, 41, 48, 57]. However, some family caregivers and practitioners considered it constituted basic human care and could not be forgone [27, 39, 55]. Compared to withholding ANH, the withdrawal of ANH was more distressing due to its more concrete association with death [49]. Furthermore, artificial hydration was more acceptable for PLWD than artificial nutrition, especially during an acute illness [33, 35, 36, 48, 49]. Views of ANH being part of basic human care were usually derived from social, religious, racial and professional values of sanctity of life among the caregivers and healthcare practitioners [27, 30, 35, 37, 39, 53, 55] especially in some cultures. For example, in Israel, Japan and USA, ANH provided hope and psychosocial benefits to the patient’s family [50, 53, 57].

- Values and preferences in making decision

Family caregivers and practitioners varied when ranking the priority of values and ethical principles to be used in the decision process [31, 52, 59]. Respect for the autonomy of the person was a common concern across the studies. Written advance care plans (ACP) were rare, but if available, they were honored by family caregivers and practitioners [37, 48, 56]. However, the written ACP or any previously stated directives might be seen as vague, outdated, and no substitute for an ongoing discussion [48, 49]. PLWD might not be able to fully understand their future when they made advance decisions [61].

If previous stated directives were absent or unreliable, family caregivers and practitioners mostly relied on the person’s presumed wishes [32, 35, 45, 47] and interpretations of their current behaviors to maintain their autonomy [25, 40, 43, 48, 49]. Interpretation of the persons’ behaviors such as facial expressions and appearing to ‘decline’ food, varied among family caregivers and practitioners, and was challenging to understand [25, 46, 57, 68]. When the person’s preferences were unclear, the values of family caregivers and social norms would override the decisions [39, 43, 47, 50, 53].

#### Consideration of feasibility

Some feeding interventions were considered impractical for certain situations or settings due to limited resources and organizational and legal restrictions.

- Micro level

When eating and drinking difficulties became severe, PLWD required a high level of care in terms of time and staffing from families, nursing home staff and hospital staff [40, 59]. Difficulties may also lead to recurrent aspiration, pneumonia and repeated hospitalizations. In these circumstances, hand-feeding could be seen as impractical, and tube feeding was needed to limit costs of care and prevent unnecessary hospitalizations [40, 44, 62]. At the individual level, family caregivers with higher perceived financial burden, caring for PLWD with poor clinical outcomes, tended to forgo tube feeding [29]. However, a quarter of family caregivers perceived that a feeding tube was inserted to make it easier for practitioners to provide nutrition and hydration for PLWD [26, 45, 50].

- Meso level

Studies reported that the amount of time and staffing for each person with dementia should be equally distributed among patients within the healthcare settings, as reasonably as possible [55, 61]. This should also not compromise practitioners’ professional integrity and dedication to care [59].

- Macro level

Legal regulations and organizational culture played an important role in increasing tube feeding, for example, where these focused on preserving the patient’s life and did not value hand-feeding [44, 55, 56]. In some countries, legal regulations offered incentives for hospitals to promote the use of tube feeding, and long-term care facilities sometimes required PEG before transfer from hospitals [38, 39, 50, 59]. Around 60% of physicians felt pressured by nursing homes or long term care facilities to perform PEG [28, 34, 38]. Physicians were often concerned about litigation if they did not start ANH [28, 56].

#### Deliberation between preferred choices and actual decision

Each decision-maker could have different preferred choices in mind towards a decision. However, as they had unequal influence on the decision-making process, some decision-makers would be precluded from making the final decision.

Physicians who advised against PEG for people with advance dementia had better knowledge about risks and benefits and were less concerned about litigation [28]. However, family caregivers and practitioners often had feelings of uncertainty about patient preferences [25, 32, 46] and their own [29, 41, 47, 54]. Some family caregivers avoided discussing feeding methods and left decisions to practitioners [50]. In a few cases, the decision-making process was almost unnoticed because it was clear what decisions should be made; either the patient’s condition was very severe or there was a clear agreement concerning further treatments [47].

Physicians were usually the final decision-makers [40, 47, 48]. On some occasions, opinions of family caregivers were also decisive [32, 34, 49]. However, when there were multiple family members, their preferred choices were sometimes conflicting [41, 47, 64] and required a key decision-maker determined either by law or closeness of relationship [53]. Nonetheless, it was common that other practitioners and even family caregivers were precluded from sharing their preferred decisions [26, 38, 40], especially in healthcare systems with a paternalistic approach where physicians hold strong influence over the decisions [39, 62]. For example, some physicians excluded nurses from decision-making, as they believed nurses relied excessively on personal and emotional factors [35, 47].

#### Implementation and outcome evaluation

Family caregivers and professionals sometimes provided nutrition and hydration despite limited support and disagreement with other decision-makers. When they found the interventions did not work, they would call for a reconsideration of the decisions with decision-makers.

- Implementation of the actual decision

Many decisions were made by an individual and immediately implemented without need for discussion. For example, practitioners and family caregivers accepted refusal to eat by the person with dementia on a day-to-day basis, but not over an extended period of time [40, 46, 52, 58]. They used *‘tricks and techniques’*, including verbal reminders, touching, pressing the mouth softly with a spoon, using of special cup, and environmental modifications [40, 46]. This was to postpone ANH decisions, which required discussion with other people.

In the context of shared decisions, once mutually agreed, the decision could be implemented either to start or to forgo ANH [40, 42, 47]. Practitioners were willing to continue hand-feeding if the family was well-informed and accepted the risk of aspiration and weight loss [44]. Long-term ANH was generally considered inappropriate for PLWD [24, 29, 43], although it was acceptable in some culture and organizations [30, 39, 44, 50, 56]. It is worth noting that ANH was often used as a temporary measure as part of treatment for acute conditions [36, 48, 49]. Practitioners performed ongoing evaluations until the person was stabilized, at which point they could either resume oral feeding [56, 65] or wait for further decision-making on implementing permanent ANH [48, 63]. On agreement to give ANH to PLWD, physicians expected artificial nutrition to be used for a longer period (few weeks) than artificial hydration (days) [36].

When practitioners and family caregivers did not mutually agree final decisions, this sometimes led to challenging opinions, including questioning, complete disagreement and refusal to enact the decision. Some nurses resisted decisions and repeatedly raised their concerns to physicians or in team meetings [40, 42, 48]. Sometimes, together with family caregivers as a group, they challenged the physicians [47]. However, for some nurses it was an unwritten rule not to speak against physicians in front of family caregivers [41]. They might personally influence the family caregivers to request reconsiderations from physicians and encourage them to take patients home without ANH [40]. In some cases, both nurses and physicians had to act against their beliefs and consciences to follow family’s requests to give ANH [34, 41, 47]. Some nurses might adapt or deliver ANH with a more tender and respectful approach [41, 55]. They also continued mouth care (use of ice cube, sparkling water and lip balm), regularly monitored the patient’s pain and position, and still offered food, if allowed [40]. These were to minimize discomfort as much as possible.

- Outcome monitoring and renegotiation

The use of ANH, especially tube feeding, might require physical or medical restraints [24, 26, 40, 55, 57, 67], thereby leading to multiple complications [26, 57, 62] and causing distress for family caregivers and practitioners [26, 41, 46, 48, 56]. When tube feeding was not effective, or its burden outweighed the benefits, there was a renegotiation between family caregivers and practitioners, which resulted in forgoing tube feeding and starting comfort feeding only [31, 37, 64, 67]. Eating and drinking decisions were often followed by nurses providing further medical explanations and psychological support for the family, and preparing them for a possible farewell [40, 47].

## Discussion

We aimed to explore the decision-making process regarding nutrition and hydration for PLWD and map this onto a decision-making model. We studied each decision-making step and attempted to understand interactions between them. For example, preferences, values and feasibility regarding certain feeding interventions may depend on information shared among all involved. This may eventually determine their preferred choices and actual decision. Decision-makers could iteratively check the steps to probe and resolve any suboptimal decision-making process. The review also emphasized that each step of the decision-making process is contextual and varied, which results in a wide range of final decisions.

### Mapping onto IP-SDM model

In this review, we extracted and developed initial codes based on the IP-SDM model. We then developed themes and revised the model to fit the decision-making process of nutrition and hydration for PLWD (as shown in Fig. 2). The original model was developed and validated in the primary care context [14, 22]. It might not be fully applicable to the dementia context, given the slow deterioration and life-limiting nature of dementia, and involvement of people lacking mental capacity. Regarding the included studies, the decisions involved practitioners and family caregivers or surrogate decision-makers acting upon the best interest of PLWD. PLWD might have indirect inputs for decision-makers to consider, such as their wellbeing and health status. In many decisions, some practitioners, such as nursing home staff, speech and language therapists and dieticians were involved only in the assessments of eating problems, but did not fully influence decision-making. Hence, our model does not support the interprofessional approach. While decisions were hugely influenced by the environment at every step, there is less evidence on consideration of feasibility at service levels (meso and macro), but more evidence at an individual (micro) level.

We found that the decisions about nutrition and hydration for PLWD were generally too complex to be mapped onto the precise linear steps of the model. We rearranged the steps in the original model to confirm that some decisions may not need shared elements of the decision-making process and some steps could simultaneously occur. For example, the actual decisions were sometimes made by a frontline stakeholder without explicitly considering the preferred choices of all involved, which challenged the shared approach. There were also everyday decisions about feeding at each meal which might have been considered small and too insignificant to trigger the shared elements of the process. However, these decisions often caused distress in decision-makers, as they were made with little support. The implementation of decisions was often immediately followed by outcome evaluation which in turn influenced the implementation. For example, nurses stopped hand-feeding or provided ANH more gently when PLWD showed signs of distress. This might also prompt renegotiation among all involved to revisit the process. We would therefore not suggest taking the steps of the model as fixed, linear or even bi-directional. Instead, steps should be viewed as cyclical and contextually dependent reminders of what to consider when making shared decisions; not every step is needed for every decision.

### Determinants of decisions

Consistent with the existing literature, we found that decision-making processes were influenced by decision-makers’ own views of nutrition and hydration interventions, perceptions of others involved and resources for decision-making [23, 69]. They tended to forgo ANH for PLWD if they were well-informed of its risks and benefits, recognized poor disease prognosis, knew the patient’s wishes to stop feeding, had negative experiences of ANH for previous family members and believed that ANH could prolong the patient’s life unnecessarily. Despite its futility, ANH was deemed indispensable due to practitioners and caregivers’ unsettling emotions regarding the decisions, emphasis on psychosocial benefits of ANH, concern about litigation and pressure from other stakeholders to start ANH, all of which are in line with previous studies [70, 71]. Unlike some other healthcare decisions that rely upon arbitrary, evidence-based scales, such as the early management of acute ischemic stroke, decisions about nutrition and hydration for PLWD may need a sensitive approach to clarify people’s opinions, feelings and values towards the decisions [23, 72].

### Facilitators and barriers of decision-making

#### Facilitators

The review found that family caregivers and practitioners had a better experience of decision-making if they received support from each other, were understood and respected in their roles in making decisions, established trust from open communications and discussions, and had a chance to renegotiate. This is in line with previous studies that found decision-making in dementia care can be facilitated by honest, trusted, respectful and shared discussions among the decision-makers [73].

#### Barriers

Decision-making by family caregivers and practitioners may be hindered by the unpredictable prognosis of dementia, unclear role and responsibilities, limited time to make decisions, conflicting opinions of involved people, unreliable advance directives, difficult interpretations of current patient’s behaviors, and social and organizational expectations. This is consistent with previous studies on decisions regarding eating problems for PLWD and some other progressive neurological disorders, such as Parkinson’s disease and multiple sclerosis [11, 74]. Indeed, these barriers could be consequences of the failure of any step in the decision-making process.

### Strengths and limitations

To our knowledge, this is the first systematic review reporting the decision-making process of nutrition and hydration for PLWD and mapping experiences to a decision-making framework. It covers a wide range of feeding methods and involves a substantial number of participants from included studies. We systematically used the IP-SDM model as a theory-driven framework to investigate the underlying decision-making process. Although this was deductive, we still allowed for results to refine and shape the theory – i.e. renaming categories and expanding on the detail and generating themes within each of these categories – hence allowing an inductive approach to the analysis. We used a detailed search strategy. Data synthesis was conducted through iterative discussions among the review team to increase the review’s robustness.

The review included many types of studies reporting different levels of evidence, which made synthesis challenging. However, we intended to include a wide range of study designs to gain an in-depth understanding enabling a focus on different areas of the decision-making process and complementing each other. We tabulated the data using a comprehensive data extraction table to ensure that all data were systematically considered (see Additional file 4). We also weighted the discussion and findings according to their quality, and then study design. Due to time and resource limitations, full-text screening, data extraction, and coding were led by a single reviewer with some piloting, which could have led to bias and errors. To mitigate this risk as far as possible, all abstracts and 35% of eligible full-texts were double screened as well as detailed study selection criteria, extraction and coding frameworks were used based on extensive, iterative discussions with the review team. Additionally, due to limited existing evidence, all included studies involved decisions for PLWD at a severe or advanced stage. The review may have limited application to decision-making for people with mild or moderate dementia. Finally, although we attempted to understand the decision-making process from various values and context in different countries, there was huge variation of organizational routines, legal restrictions and social values. It was impossible to reflect the decision-making process that applies to every individual case or every context, but we were able to show the overview of such process within the included studies.

### Implications for future research, policy, and clinical practice

This review has identified the necessary steps in making decisions regarding nutrition and hydration for PLWD; it can guide healthcare practitioners and policy makers on what issues they should be aware of, how and when to address the issues in the decision-making process and whom they should involve in the decisions. In the later stages, family caregivers and practitioners can be facilitated with decisional support, for example, a decision aid which has been found to increase the level of knowledge, improve quality of communication and reduce decisional conflicts [75]. This review helps to guide the focus of decisional support and identify mechanisms to overcome many of the identified barriers. It should deliver sufficient information about the interventions, help clarify people’s opinions and values, and provide educational training and support to those involved [76]. Healthcare and social policy should be carefully devised to acknowledge its influences on decision-making. Future research may explore the decision-making process of other healthcare decisions with the same approach as this review. From our results, it would be interesting to investigate underlying assumptions of the different attitudes regarding artificial hydration and artificial nutrition as basic care.

## Conclusions

The decision-making process regarding nutrition and hydration for PLWD is complex and does not follow a linear process. It needs an informed, value-sensitive, and collaborative process with explicit roles of all involved. However, the decisions are usually considered with unclear procedures and with a lack of support. Decisional support is needed and should be approached in a shared and stepwise manner.

## Supplementary Information

**Additional file 1:.** PRISMA _checklist.docx

**Additional file 2:.** Manual guide_for study screening.docx

**Additional file 3:.** Full search strategy MEDLINE.docx

**Additional file 4:.** Full Data Extraction Table.xlsx

**Additional file 5:.** Overall judgements of quality assessment.docx

## Data Availability

All data generated or analyzed during this study are included in this published article and its supplementary information files.

## Abbreviations

ANH: Artificial nutrition and hydration
PEG: Percutaneous endoscopic gastrostomy
CFO: Comfort feeding only
PLWD: People living with dementia
PRISMA: Preferred reporting items for systematic reviews and meta-analysis
MeSH: Medical subject heading
CASP: Critical appraisal skills programme
IP-SDM: Inter-professional shared decision making
ODSF: Ottawa Decision Support Framework
NG: Nasogastric
IV: Intravenous
VSED: Voluntary stopping eating and drinking
TPN: Total parenteral nutrition
RIG: Radiologically inserted gastrostomy
ACP: Advance care plans
