# Supplementary material for: Mapping and understanding the decision-making process for providing nutrition and hydration to people living with dementia: a systematic review
Source: BMC Geriatr. 2020 Dec 2;20:520. doi: 10.1186/s12877-020-01931-y (PMC7709405; doi:10.1186/s12877-020-01931-y)
Supplement: Supplementary file 2 — Additional file 2:. Manual guide_for study screening.docx [file 12877_2020_1931_MOESM2_ESM.docx]

**Additional file 2: Manual guide for study selection of systematic review**

**Overview of inclusion and exclusion criteria**

| **Inclusion** |
| --- |
| - Peer-reviewed paper focusses or makes reference to a process and/or its determinants of decision making on nutrition and hydration for people with dementia - All study designs including quantitative study, qualitative study, case report - Involved or aimed at people with dementia, formal or informal caregivers, and health professionals - No restrictions on year of publication |
| **Exclusion** |
| - Commentary, editorial and opinion pieces - Conference abstracts - Study or review protocol - PhD or other qualification theses - Non-English language |

***Additional guides***

1. Nutrition and hydration choices/interventions for people with dementia (PWD) would be either
   1. Oral/hand-feeding (e.g. comfort feeding, modified food like finger food and nutrient-dense food, and any managements to maintain PWD to eat/drink by oral like laying the table together and adapted cutlery etc.) OR
   2. Artificial nutrition/hydration interventions (e.g. enteral/tube feeding, parenteral feeding/hydration, intravenous fluid, hypodermoclysis etc.) BUT
   3. Withholding/ withdrawal/ forgo of those would be ‘one’ of the choices.
   4. It is difficult for caregivers and professionals to choose (make a decision) among these choices > we are looking at how they choose (i.e. make the decision).
2. Include papers that studied on a **description/ process/ determinants/ facilitators/ barriers/ outcome/ effectiveness of**
   1. Decision making *and/or*
   2. Decision intervention/aid on nutrition/hydration for PWD
      1. e.g. education of existing choices, the Fact Box decision support tools, a co-design heuristic tool on end of life care for PWD
3. **RULE OF PRIOR OR DURING** the decision-making process. If the phenomena of interests in the study was about after the decision, then it might not decision making.
   1. e.g. excluded a legal case/court judgement, an evaluation of certain decisions/intervention, an opinion after using certain intervention – it would be an ‘after’.
   2. Unless the ‘post hoc event’ became, in turn, a factor in re-visiting a decision making in that case or among the participants of the study. We will include that study.


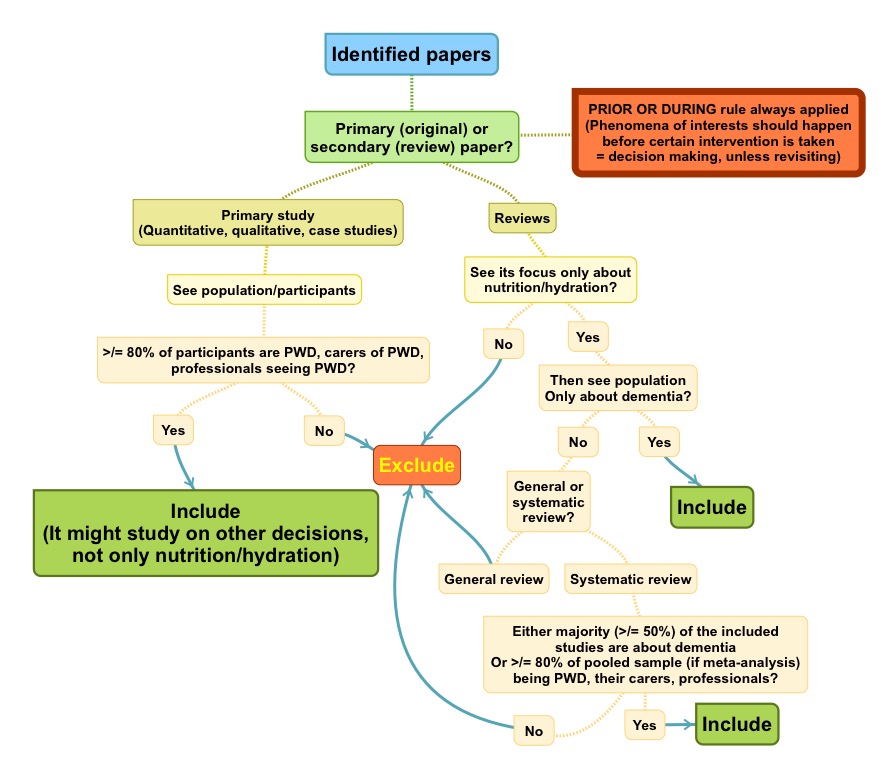


1. Focused population of potential studies
   1. Specific only to dementia – yes (i.e. PWD, caregivers, professionals)
   2. Not specific to dementia
      1. Primary/ original research (quantitative, qualitative, case studies) – If applicable and information available, the cut-off of at least 80% of the sample having dementia and/or being caregivers/professionals of PWD can be applied. This cut-off was based on proportion of PLWD as nursing home residents and used by other published studies in Cochrane [[1](#_ENREF_1), [2](#_ENREF_2)].
         1. This also applies to a study that PWD and/or caregivers-professionals of PWD being asked to respond to the hypothetical situation of making a decision on nutrition (e.g. using vignettes) – **BUT** excluded studies that surveyed among healthy older people participants or equivalent.
         2. Case studies – normally, it should be about dementia patient(s) only
            1. If not, e.g. they may present the cases of dementia, PD and stroke in a single study. Just include it. We would pick up only a case of demented patient when extracting data.
      2. General reviews **(just for reference search)**
         1. only dementia – yes
         2. If not, no; It is really difficult to judge that an individual paper and/or certain statement was substantially discussed or drawn based on PWD information.
      3. Systematic review **(just for reference search)**
         1. Yes, if either majority (>50%) of included studies are about dementia or >/= 80% of pooled sample (if meta-analysis) being PWD, caregivers of PWD, health professionals caring PWD.
         2. No, if the data is unavailable OR not reach the above cut-off.
2. Focused topics of potential studies
   1. Primary/original research – quantitative, qualitative, case studies
      1. Include the original studies that make a reference, but not limit, to the decisions on nutrition and hydration (i.e. they may also study on other decisions for PWD at the same time, e.g. antibiotic, DNR, placement).
   2. Reviews/systematic reviews **(just for reference search)**
      1. Include only studies that focused on nutrition/hydration decisions
3. Exclude papers that studied on the description/outcomes/effectiveness of a certain type of nutrition/hydration intervention, but not explicitly on the decision making. – **may refer to No. 4**
   1. e.g. effectiveness of PEG, modified food for PWD
4. Exclude papers that simply described existing choices of nutrition and hydration for PWD, but not explicitly about the decision making on the choices.
5. Exclude guidelines, and government or organizational recommendations, as these will be informed by existing empirical studies, academic reviews and case studies anyway.
6. Exclude a legal case study/ court report. As it will be a review of the case regarding legal judgement. Rather than on the decision making process at the time of making that decision. (may refer to No.4)
7. Exclude an ethical study; if it is merely a philosophical explanation/standards and did not take into account in making a decision.
   1. We would interest in individual level or actual decision making process, i.e. include the study of participants of the study making a decision based on ethical consideration. AND/OR the ethical consideration was explicitly applied to the decision making in the participant(s) or case(s) of the study.
8. Exclude advance care planning (ACP) intervention or study. As it could be a cognitive and pre-planning, and might not actually involve at the ‘time/situation’ of making the decision. Unless the studies explicitly refer that the ACP was applied directly into a ‘real decision-making situation’ and specifically on ‘nutrition and hydration’. If the case we will include it.

**References**

1. Helvik AS, Engedal K, Benth JS, Selbaek G. Prevalence and Severity of Dementia in Nursing Home Residents. Dementia and geriatric cognitive disorders. 2015;40(3-4):166-77. doi: 10.1159/000433525.

2. Murphy E, Froggatt K, Connolly S, O'Shea E, Sampson EL, Casey D, et al. Palliative care interventions in advanced dementia. Cochrane Database Syst Rev. 2016;12:CD011513. doi: 10.1002/14651858.CD011513.pub2.
