# Supplementary material for: Mapping and understanding the decision-making process for providing nutrition and hydration to people living with dementia: a systematic review
Source: BMC Geriatr. 2020 Dec 2;20:520. doi: 10.1186/s12877-020-01931-y (PMC7709405; doi:10.1186/s12877-020-01931-y)
Supplement: Supplementary file 3 — Additional file 3:. Full search strategy MEDLINE.docx [file 12877_2020_1931_MOESM3_ESM.docx]

**Additional file 3: Full electronic search strategy for MEDLINE database**

| **No** | **Search strategy** | **Hits retrieved** |
| --- | --- | --- |
| 1 | dement$.mp. | 126912 |
| 2 | alzheimer$.mp. | 155024 |
| 3 | exp Dementia/ | 161107 |
| 4 | Alzheimer Disease/ | 91168 |
| 5 | exp Nutritional Support/ | 44420 |
| 6 | Enteral Nutrition/ | 19314 |
| 7 | exp Parenteral Nutrition/ | 23718 |
| 8 | (nutrition$ adj3 support$).mp. | 16210 |
| 9 | (enteral$ adj3 (feed$ or nutrition$)).mp. | 25794 |
| 10 | (artificial$ adj3 (feed$ or nutrition$)).mp. | 3421 |
| 11 | Intubation, Gastrointestinal/ | 9575 |
| 12 | (tube adj3 feed$).mp. | 7133 |
| 13 | (percutaneous adj3 (feed$ or nutrition$ or tube)).mp. | 1630 |
| 14 | (endoscop$ adj3 gastrostom$).mp. | 3426 |
| 15 | (gastrostom$ adj3 tube).mp. | 2785 |
| 16 | (stomach adj3 (feed$ or nutrition$ or tube)).mp. | 1353 |
| 17 | (nasogastric$ adj3 (feed$ or nutrition$ or tube)).mp. | 5348 |
| 18 | (forc$ adj3 feed$).mp. | 2271 |
| 19 | (parenteral$ adj3 (nutrition$ or feed$ or tube)).mp. | 32281 |
| 20 | (feed$ adj3 method$).mp. | 5321 |
| 21 | (comfort$ adj3 feed$).mp. | 111 |
| 22 | (oral$ adj3 (feed$ or nutrition$)).mp. | 7292 |
| 23 | (hand$ adj3 feed$).mp. | 1126 |
| 24 | (modif$ adj3 (feed$ or food$)).mp. | 5073 |
| 25 | nutrition$.mp. | 375693 |
| 26 | exp fluid therapy/ or home infusion therapy/ | 20502 |
| 27 | Dehydration/ | 13079 |
| 28 | Drinking/ | 14087 |
| 29 | hydration$.mp. | 34825 |
| 30 | (artificial$ adj3 hydration).mp. | 469 |
| 31 | fluid$.mp. | 602852 |
| 32 | hypodermoclysis.mp. | 217 |
| 33 | decision making/ or choice behavior/ or consensus/ or exp "dissent and disputes"/ or uncertainty/ | 147166 |
| 34 | clinical decision-making/ or medical futility/ | 10641 |
| 35 | Decision Support Techniques/ | 19771 |
| 36 | Decision Support Systems, Clinical/ | 7675 |
| 37 | decision$.mp. | 432533 |
| 38 | judgement$.mp. | 15132 |
| 39 | (choice$ adj3 (behavio?r* or informed or making)).mp. | 39432 |
| 40 | exp Withholding Treatment/ | 15003 |
| 41 | 1 or 2 or 3 or 4 | 250936 |
| 42 | 5 or 6 or 7 or 8 or 9 or 10 or 11 or 12 or 13 or 14 or 15 or 16 or 17 or 18 or 19 or 20 or 21 or 22 or 23 or 24 or 25 or 26 or 27 or 28 or 29 or 30 or 31 or 32 | 1047179 |
| 43 | 33 or 34 or 35 or 36 or 37 or 38 or 39 or 40 | 507759 |
| 44 | 41 and 42 and 43 | 568 |
| 45 | limit 44 to English language | 533 |

**Hits retrieved:** 533 (29 January 2020)
