# Supplementary material for: Mapping and understanding the decision-making process for providing nutrition and hydration to people living with dementia: a systematic review
Source: BMC Geriatr. 2020 Dec 2;20:520. doi: 10.1186/s12877-020-01931-y (PMC7709405; doi:10.1186/s12877-020-01931-y)
Supplement: Supplementary file 5 — Additional file 5:. Overall judgements of quality assessment.docx [file 12877_2020_1931_MOESM5_ESM.docx]

**Additional file 5: Overall judgements of quality assessment of included studies**

| **First author (year)** | **Summary notes for quality assessment** |
| --- | --- |
| Aita K (2007) | GOOD: clear and justified objectives. Suitable methodology (grounded theory -seeking for theoretical explanation, ongoing analysis, theoretical sampling) to explore experiences and attitudes. Well-planned and clear participant selection and recruitment (maximize variation). Fairly good interview questions. Data saturation was reached. Well-planned and clear data analysis, use of multiple methods to enhance creditability (more than one analyst, respondent validation). Relevant and very important findings. |
| Babiarczyk B (2014) | POOR: Poor defined selection of participants, insufficient participants, limited data collection and analysis, lack of limitations acknowledgement |
| Back AL (2005) | MODERATE: This case study described the decision-making process quite clearly and is supported by relevant literature. However, it seemed not focused on the case, rather on theoretical discussions. Sometimes they used hypothetical situations. This could create confusions as if the discussions applicable to the case. The case was quite well-described. Family and physician’s perspectives were discussed. It was unclear what the latest conditions of the case looked like. |
| Berger JT (1996) | GOOD: well-description of the case and process. Comprehensive and holistic discussion of the decision-making process. Social factors, nursing home restriction, perspectives were described and discussed. |
| Bryon E (2010), (2012a), (2012b) | GOOD: well-designed study including settings and participants selection, data analysis (method triangulation, audit trail, ongoing data collection and analysis, constant compare and contrast), well-formed aims and research questions, well-described findings. |
| Buiting HM (2011) | MODERATE: Well-formed research questions, objectives, Poor participant selection system - worried about the differences of participants of the two countries- may effect the interpretation of findings especially when aiming to determine cultural effects - In Dutch: physicians with nursing home medicine training vs Australian GPs and geriatricians i.e. the differences may be from differences background of training not purely culturally. Well-designed data analysis process. Relevant findings. |
| Chen PR (2019) | MODERATE: Clear objectives with suitable methodology. Great sample size, but unclear about recruitment. No non-response rate was reported. Well-designed questionnaire, but difficult to interpret sum-score or weight among the items and could have missed institutional and social factors. Response agree/disagree to each item may not directly link to the reduction of tube feeding use. Good analysis plan with adjustment, but small sample size for subgroup analysis. Relevant and useful findings. |
| Christenson J (2019) | GOOD: The case and situations were clearly described. The process of ethical meeting/ consideration was clarified. Family’s opinion, previously stated wishes of the patients, past eating management and current patient’s status were taken into account. |
| Clibbens R (1996) | POOR: Not much details of the case and process of consultation/ decision-making. Typical scenarios/ situations. Useful conclusions/recommendation. |
| Gieniusz M (2018) | MODERATE: Clear objectives (but may be not directly about the review). Despite very low response rate (combined response rate = 17%), well-planned recruitment method (Tailored Design Method (TMD) - to increase response rate) - subjected to non-respondent bias. Suitable research methodology with well-designed questionnaire (expert reviews, iteration and pilot). No adjustment for confounding factors. Relevant and useful findings. |
| Gil E (2018) | MODERATE: Good aims and research questions fit to the research methods chosen. Concerns over the recruitment and selection bias-feeling obligated and only those who favour PEG included. This could lead to bias of the findings. Not mentioned about data saturation. Data triangulation with multiple methods (observations and interviews). Good findings' presentation -concise and relevant. Good implication. |
| Golan I (2007) | POOR: Clear objectives with suitable methodology (questionnaire with tailored for each group). Participants from four hospitals - identified from PWD who were referred for PEG, potentially with certain clinical conditions. Many possible confounding factors (no adjustment) and selection bias. Relevant and make-sense findings. |
| Hodges MO (1994) (Quantitative) | GOOD: Clear aims and research questions, Suitable methodology (questionnaire with case scenarios). Fairly well-planned recruitment and selection system (excluded those not enough personal experiences with TF) - relatively low response rate (58.1%). Questionnaire was developed over a year and two-regional pretested. Good data analysis (suitable and clear). Tested for age and gender. Relevant and important finings. |
| Hodges MO (1994) (Case study) | POOR: No information of the case (very limited) and final decisions/ actions were presented. Just discussion and recommendations of the authors. Relevant and representative case and situations, especially about use of restraints. |
| Jansson L (1992) | POOR: clear aims and research questions. Suitable methodology (interviews). Well-structured interviews using case scenario. Concerns about recruitment and selection: no criteria - self-defined by manager. Unclear data analysis process (relatively short). No discussion of potential bias and influence of the researchers' role. No data saturation was mentioned. Relevant findings. |
| Jox RJ (2012) | GOOD: Clear objectives. Appropriate methods (think aloud - avoid rationalization of interviews). Well-designed case scenarios with variations. Well-planned data analysis (content analysis). Relevant and insightful findings (good presentation) |
| Kuehlmeyer K (2015) | GOOD: Clear objectives and research questions. Suitable methodological methods. Good recruitment (stratified of nursing home) and Relatively good participant selection procedure. Well-designed questionnaire (consult with experts and pre-test). Appropriate data analysis. Relevant and important findings. Acknowledged multiple limitations of the study. |
| Kwok T (2007) | MODERATE: Clear objectives and research questions. Suitable methods (questionnaire). Worry about the before and after were just immediate assessed - real practice, not only provide information and then effect the decisions (multiple factors) – might feel needed to follow the provided evidence. Various sources of participants. 57% response rate - not high, may bias. Relevant and important findings. |
| Lopez RP (2010) | MODERATE: clear objectives. Suitable method (in-depth interview). Unclear of use topic guide (but shown some topics questions. Not convincing recruitment methods (unsure how to gain various perspectives as said). Data collection continued until themes saturation (but this was under a parent study?). Relatively good data analysis. Relevant and important findings. |
| Lopez RP (2010) | GOOD: Clear objectives and research questions. Suitable methods. Good and justified recruitment and selection methods. Well-planned data collection (use of multiple qualitative methods). Well and rigorous data analysis. Well acknowledgement of bias, limitations. Relevant and important findings. |
| Luhnen J (2017) | GOOD: clear objectives. Suitable methods (interviews). Well-designed topic guide (piloted, amended), Fairly good data analysis plan. Extensively discussed on its limitations and bias. Relevant and important findings. |
| Meier CA (2015) | GOOD: well-described of the case (characteristics, problems, context), decision-making process and discussion. Relevant and important findings. |
| Modi SC (2007) | MODERATE: Well-defined objectives and rationale. Suitable methods. Fairly good questionnaire design - expert reviews, but unsure about the use of scenarios which annotated about 'race' only one word - can determine effects of race in decisions. Fairly justified to combine - recommend against PEG with no recommendations as default mode (which default may mean recommend as well for some physicians); Good analysis - controlled for confounding factors. Relevant and interesting findings. |
| Nagao N (2008) | POOR: Clear Objective. Suitable methodology. Only 4 experts with some having limited experiences. Questionable about conclusion regarding to comparison between the two countries. Quite good data collection methods - using variety of methods - questionnaire, interview (guided by the findings of the questionnaire - not so appropriate), team case study and then follow up interview). Fairly good data analysis (only multiple analyst was used, not mentioned about other creditability). Relevant but questionable findings. Critically - Need more participants. |
| Norberg A (1987) (Sweden study) | POOR: Clear objectives. Suitable methodology (qualitative). Quite not sure about how the participants (the longest working) were approached (by who). No discussion about the influence of the role of the authors, any possible bias, ethical considerations. No information about origins of the topic guide. Quite unsure about quantified qualitative data as they would depend on questions and probe at each interview. No other methods to increase rigor, except multiple analysts. Relevant and important findings. |
| Norberg A (1987) (Israel study) | POOR: clear objectives. Suitable methodology. Fairly good participants selection and recruitment. Unclear about the of interviews (both individual and group) - no information about topic guide, how to develop the topics. Very limited data analysis process (by who and how). Relevant and important findings. |
| Norberg A (1994) | MODERATE: Well-defined objectives. Quite suitable methodology - structured interviews then coding and counting use statistical analysis. Unclear selection of institutions and participants (no clear criteria and justification presented). Low number of participants per countries and may subject to have influence of cultural and health care system variation. No adjustment. Relevant and important findings. |
| Orr RD (1991) | GOOD: Well-descriptions of the cases, decision-making process and up to the implementation of decisions (not taking Ethics consultation and consequences of providing TF) |
| Orr RD (2002) | GOOD: well-description of the case concerning health conditions, context (religious, past history), attitudes of the PWD (previous wishes/ wills), family and staff. Well-defined decision-making process. Enough follow-up time. |
| Pang MCS (2007) | POOR: Clear objectives. Suitable methodology. Unclear selection of sites and participants. Unclear recruitment methods. Unclear who and how they were included. Unclear about the data collection - who and how they were observed. No information of data analysis. Relevant and important findings - but presenting the findings together with literatures makes this part confusing. (checked for references). |
| Pasman HRW (2003), (2004a); The (2002) | GOOD: Good background leads to clear and justified objectives and research questions. Suitable methodology (justified to use of observations. Good setting and participant selection. Triangulation - observations, interviews, filed notes, access to medical and nurse record. Very rigour (two analysts, collecting then analysing then re-collecting data to validate). Good analysis plan. Well-concerned of ethical issues. Relevant, clear and important findings. |
| Pasman HRW (2004b) | MODERATE: Clear and logical objectives. Suitable methodology - to compare. Concerns over the participant selection (identified by the NHP) may affect the results especially when exploring the 'participation' of decision-making process per se. Data analysis- quite descriptive statistics, no adjustment - unclear how to interpret and apply the results. Relevant and interesting finding. |
| Pengo V (2017); Valentini E (2014) | MODERATE: clear objectives. Suitable methodology - use of structured questionnaire to compare and find correlations. High attrition rate may lead to non-respondent bias plus member bias. Quite well-designed questionnaire. Fairly good data analysis but no adjustments for many potential confounding factors. Relevant and important findings. |
| Scarpinato N (2000) | POOR: Unclear objectives and focus. Quite not a good description of the case, rather focus on opinions and ideas. Relevant findings concerning a factor of depression. |
| Shega JW (2003) | MODERATE: Clear and logical objectives. Suitable methodology. Hight attrition but tests showed no differences of non-respondents. Well-designed questionnaire (piloted at two universities). Fairly good analysis but no adjustment. Quite extensive discussion about potential bias but not confounding factors. Relevant and important findings. |
| Smith L (2016) | POOR: Well-defined and logical objectives. Fairly poor recruitment methods (17/27 those non-respondents may hold different views), unclear criteria. Well-designed interview questions (expert reviewed). Unclear data analysis process, mostly referred to theoretical framework. No influence of role and potential bias discussed. Not many vigorous methods to increase creditability were discussed. Relevant and interesting findings. |
| Tapley M (2014) | GOOD: The case and situations were clearly described. The process was clarified. Attitudes and past history were taken into account. Multiple factors were mentioned. |
| Teno JM (2011) | GOOD: Clear and justified objectives. Suitable methodology to compare across the five states and PWD status. Well-planned recruitment methods with quite high response rate and acknowledged of recall bias. Reasonable of site selection. Well-designed of questionnaire (modified version of previously used questionnaire). Well-planned data analysis with multiple methods to adjust and control for bias and confounding factors (weighted and adjusted). Relevant and very important findings. |
| van Wigcheren PT (2007) | MODERATE: Clear and logical objectives. Suitable methods -questionnaire to compare across the decisions (food and fluids vs fluids only). Postal questionnaire - high response rate but subject to non-respondent bias (analysis showed slightly differences for sex and age group). Questionnaire about recent case in 1-year - subjected to recall bias. No adjustment in data analysis. Tried weighted to incidence calculation. Relevant and interesting findings. |
| Wilmot S (2002) | POOR: clear and logical objectives. Suitable methodology (to explore subjective experiences and attitudes, also facilitate discussion via group. Concerns over the participant selection - three focus group from three wards within one hospital, pre-existing group - may also limit discussion, not sure about the variety of views, no data saturation was discussed. More than one analyst and respondent validation. Not discussed about the influence of their role and other possible bias. Relevant and important findings. |
